# Colonization of Artificial Substrates by Invertebrate Macrofauna in a River Ecosystem—Implications for Forensic Entomology

**DOI:** 10.3390/ijerph20042834

**Published:** 2023-02-06

**Authors:** Aleksandra Bartkowska, Tomasz Mieczan, Wojciech Płaska

**Affiliations:** Department of Hydrobiology and Protection of Ecosystems, University of Life Sciences, Dobrzańskiego 37, 20-262 Lublin, Poland

**Keywords:** forensic biology, macroinvertebrates, substrate exposure, habitat conditions, adaptations

## Abstract

Forensic entomology includes the analysis of organisms colonizing various parts of the body in order to determine the circumstances of an incident, mainly the time, place, and cause of death. The presence of insects and other arthropods on carcasses can be a source of knowledge for the judicial system. However, this type of research (on submerged bodies) is less published. The aim of our study was to analyse the qualitative and quantitative structure of macroinvertebrates colonizing potential evidence in an upland river. The experimental research involved an eight-week exposure to articles of clothing made of different materials: natural materials (bottom sediments with plants from a river), synthetic (socks), and cotton (t-shirts). Control samples of water after 2, 4, 6, and 8 weeks were taken from experiment locations in the River Bystrzyca with a tube apparatus and hand net. The results indicated that the abundance of organisms on a given substrate depended on the period of development of invertebrate macrofauna and the time of exposure of the substrates. The abundance of aquatic macrofauna on the exposed items increased in direct proportion to the duration of the experiment, which may indicate the adaptability of these organisms to new habitat conditions. Among the taxonomic groups used in forensic entomology, Diptera, Coleoptera, and Odonata were the most abundant. The remaining taxa (including Heteroptera), though not widely used in judicial proceedings, can also provide valuable information about the circumstances of an incident.

## 1. Introduction

Forensic entomology is an area of science applied all over the world in criminal investigations. It combines knowledge from the fields of forensics, medicine, and the natural sciences. In many cases, the relationships between the presence of arthropods on the body and the circumstances of the crime are analysed in order to establish the time, place, and cause of the victim’s death [1]. Forensic entomology was used as early as 700 years ago. Its first application was noted in the case of an attempt to solve the case of a murder in the vicinity of rice fields. The victim had distinct incised wounds. It was assumed that they had been made with a sharp object and that this was most likely a tool used by workers. They presumed that blood would remain on the murder weapon and attract insects [2]. Aquatic organisms were first used in this field of science in Italy in 1888, when a relationship was demonstrated between small lesions on a corpse submerged in water and the presence of crustaceans feeding on it [3].

The main branch of forensic entomology is the science of using necropagous insects (taxa that colonise dead tissues) to determine or prove the time, place, and cause of an incident [4,5]. An important concept is PMSI (the postmortem submersion interval), the time between when a body is put in the water and when it is recovered. At every stage of decomposition, corpses are a very good living environment for necrophages. Organisms for which dead tissues are an ideal environment in terms of food sources and opportunities for development leave entomological evidence, which can provide important information for law enforcement authorities [6,7]. The evidence is mainly used to determine the victim’s time of death. Two methods of qualitative analysis are used for this purpose: developmental and successional. The first involves analysis of the stage of development of arthropods on the corpse, while the other analyses different species based on a successional pattern that is dependent on the decomposition stage of the corpse [5,8,9]. The place of an incident is determined using quantitative methods, which provide information on necrophagous species [10]. In their various stages of development, aquatic invertebrates present on a corpse can accumulate toxins, heavy metals, or chemical agents. This information can help law enforcement authorities determine the cause of death and the circumstances of the incident [6,10].

Aquatic fauna significantly affects the decomposition of the body. There has been little research on the colonisation of corpses by invertebrate macrofauna [11,12,13]. Studies on this subject indicate that organisms colonize the body both below and above the water level. The fauna on bodies submerged in water consists mainly of invertebrate arthropods, whose abundance is closely correlated with the stage of disintegration of the cells of the body [10]. A corpse submerged in water decomposes much more slowly than on land, where it is exposed to factors such as high temperature or air. Decomposition in fresh water and saltwater is similar. A significant factor distinguishing these environments may be the water temperature, which is a crucial element influencing the rate of cell disintegration. Post-mortem changes or injuries characteristic of individual species may also indicate the environment of the body’s decomposition [6,14]. In the case of rivers, determining where the victim is located can be difficult. The velocity of the watercourse, which depends on the slope of the land and the mass of the water, often makes it difficult to determine where the incident took place [10,15,16].

Decomposition processes are conducive to the colonization of corpses by the larvae of non-biting midges (Chironomidae), black flies (Simuliidae), mayflies of the family Heptageniidae, or caddisflies of the family Hydropsychidae [17]. These are the first organisms to appear on the body [18]. As the decomposition of the body progresses, the abundance of flies decreases and that of beetles increases. The larvae of stoneflies (Plecoptera), clown beetles (Histeridae), and carrion beetles (Silphidae) appear as well [14]. The presence of flies can be observed during putrefaction processes taking place above the water surface, while the larvae of non-biting midges are found below the water. Floating bodies are also colonised by predatory organisms or crustaceans, echinoderms, gastropods, or fish [10]. 

The experiment can thus serve as a source of information with respect to a potential crime, including the time it was committed and its course. Extensive analysis of the actions of perpetrators of a crime after it has been committed indicates that they attempt to cover their tracks, e.g., by hiding evidence in a river. Owing to the dynamic development of areas of science and various techniques that can help to detect crimes, nearly anything the perpetrator has had contact with, even indirectly, can serve as a source of information about this individual. Objects, such as the victim’s clothing, jewellery, or the implement used in committing the crime, can provide information about the criminal. 

The main goal of the research was to determine the composition of invertebrate macrofauna colonizing individual articles of clothing that were exposed for various time periods in a river ecosystem. Correlations between the density of the identified organisms and the time of colonisation of the substrates were determined. 

## 2. Materials and Methods

### 2.1. Location of the Study Area

**Figure 1:**
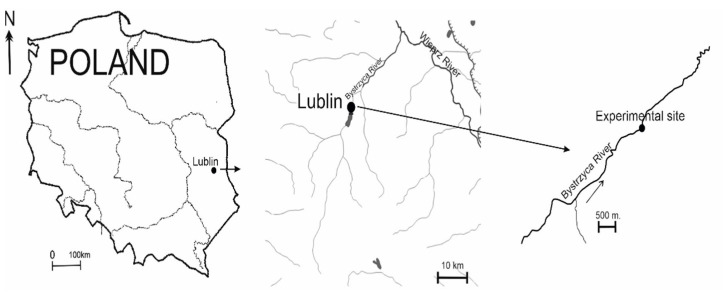
Location of the study area.

Colonization processes were studied in the River Bystrzyca in the city of Lublin (eastern Poland, 51° N, 22° E) (Figure 1). The total length of the river is 74 km, and the catchment area is 1315.5 km^2^. The Bystrzyca flows from the slopes of Roztocze at an elevation of 238 m a.s.l. and empties into the River Wieprz at an elevation of 152 m a.s.l. The average speed was 0.3 m/s. In the valley, the river bottom is made up of muck, peat, alluvial soils, and black soils, and in the remaining parts, it is composed of loess soil. The Bystrzyca flows from the south to the northeast, alongside arable fields and forests. River courses are not fed by groundwater resources. The water in rivers comes from the upper part of the catchment. The Bystrzy belongs to the category of very polluted rivers. Its waters are below all quality classes. Sources of river pollution include industrial and municipal effluents. This affects the species composition of organisms [19].

### 2.2. Course of the Experiment 

The field experiment involved the colonization of articles of clothing by invertebrate macrofauna. The clothing was made of both synthetic and natural materials: rayon socks (12) and cotton t-shirts (12). The clothes were put to use. The rayon socks used in the experiment were the same size. Cotton t-shirts were medium-sized. The control sample was bottom sediments (bottom sediments with rush plants-*Sagittaria sagittifolia* L. from the river). Each article of clothing was fastened to a rope (1 m), which was then tied to a wooden stake and placed in the river by hammering the stake into the bottom. This prevented the articles of clothing from moving with the current. Each set included 3 t-shirts and 3 socks. Macroinvertebrates colonizing the substrates submerged in the water were sampled after 2, 4, 6, and 8 weeks (Table 1). The experiment was begun on 8 May 2021 and completed on 3 July 2021. The substrates were collected by gently pulling the rope and placing the materials in plastic containers. Within a few hours, in laboratory conditions, all invertebrate organisms were carefully isolated using forceps and placed in glass Petri dishes. Each sample was placed in a labelled (date, type of material) plastic container and preserved with 80% ethyl alcohol.

Control samples of water were taken from experiment locations in the River Bystrzyca. The samples were taken using a tube apparatus (diameter 19.6 cm^2^, Limnos Sediment Sampler) and a hand net (Limnos). Invertebrate macrofauna was isolated from bottom sediments with rush plants. Using forceps, all invertebrate organisms were carefully isolated from the control material and placed in glass Petri dishes. These were placed in a labelled plastic box and preserved with 80% ethyl alcohol.

Taxonomic identification of the macrofauna colonizing the exposed substrates and determination of the exact numbers of individuals of the taxa were performed using a stereo microscope (Nikon SMZ800) and a key for the identification of aquatic macroinvertebrates [20,21]. The abundance of macrofauna was expressed per m^2^ of the substrate surface.

### 2.3. Statistical Analysis

The relationships between abundance and exposure time were analysed using SPSS software (ver. 26). Kendall’s tau correlation, a measure of the relationship between two variables, was calculated. This method determines the difference between the probability that the two variables will be ranked in the same order and the probability that they will be ranked the reverse order. Kendall’s tau correlation is analysed by calculating two variants of tau (tauB and tauC) [22].

A correct interpretation of the data requires a null hypothesis (H_0_), which postulates the absence of a relationship between variables, and an alternative hypothesis (H_A_), which indicates that such a relationship exists. The correlation coefficient (p) is interpreted in terms of statistical significance [22].

The analysis was performed in the form of correlations between two variables and presented in scatter plots. The normal distribution of the data was analysed, and taxa that did not satisfy the assumptions were excluded (*p* < 0.5).

## 3. Results

### 3.1. Taxonomic Composition

The presence of three taxonomic units of aquatic organisms was noted on the colonised substrates. Arthropods included crustaceans and insects. The representatives of Crustacea were amphipods (Amphipoda). Insects included dragonflies (Odonata), true bugs (Heteroptera), beetles (Coleoptera), and flies (Diptera). Annelids (Annelida) were represented by leeches (Hirudinea). The last group was molluscs, represented by gastropods (Gastropoda). The results of the experiment clearly show that the exposure time did not cause significant changes in the taxonomic composition. After two weeks of exposure on both cotton and synthetic substrates, the taxa observed were Amphipoda, Coleoptera, Hirudinea, Chironomidae, and Tubificinae. Four weeks after the start of the experiment, Amphipoda, Coleoptera, Odonata, Chironomidae, Tubificinae, Gastropoda, and Heteroptera were isolated in the samples from the synthetic and natural substrates. After six and eight weeks of exposure, there were no dragonflies. Amphipoda, Tubificinae, Chironomidae, Coleoptera, Gastropoda, Heteroptera, and Hirudinea were found in natural materials. In the control samples, the taxonomical compositions of macroinvertebrates from river bottom sediments differed slightly from the experimental variants.

### 3.2. Abundance of Macroinvertebrates

**Figure 2:**
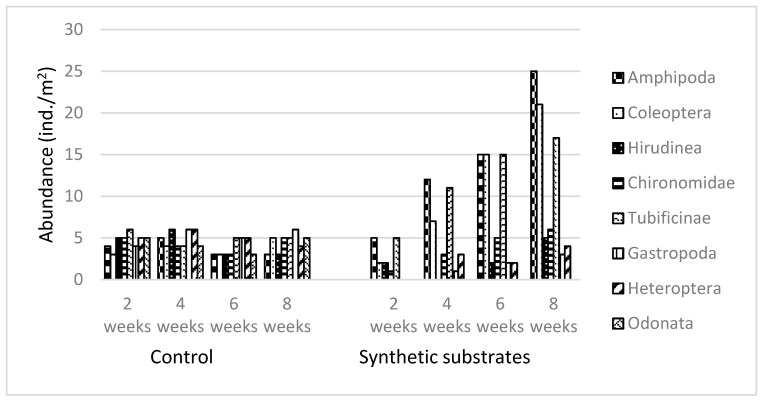
Abundance of aquatic invertebrates (ind./m^2^) on synthetic substrates and in the control samples.

The abundance of individual taxa was very similar after 2, 4, 6, and 8 weeks. On the natural materials, all taxa represented constant abundance. On the synthetic substrates, the highest abundance was noted for Amphipoda. Other taxa represented in very high numbers were Tubificinae, Chironomidae, and Coleoptera. The abundance of these four taxa increased in direct proportion to the exposure time. Small numbers of leeches (Hirudinea) and true bugs (Heteroptera) were also detected on the substrates. The abundance of macrofauna was calculated per m^2^ of substrate surface. Abundance of macroinvertebrates showed marked variation between substrates. (Figure 2).

**Figure 3:**
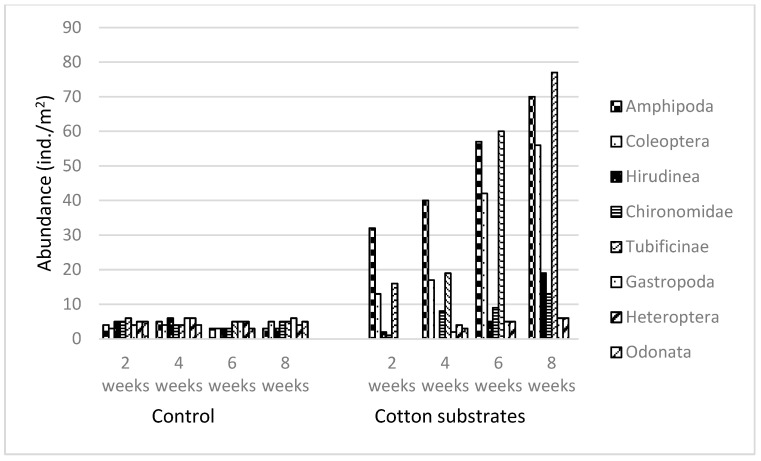
Abundance of aquatic invertebrates (ind./m^2^) on cotton substrates and in the control samples.

On the bottom sediments, all taxa represented a very similar abundance. The abundance of individual taxa on the cotton substrates was higher than that noted on the synthetic substrates. Here too, the largest group was Amhipoda, and their abundance increased in direct proportion to the exposure time. Tubificinae and beetles (Coleoptera) were numerous as well. The samples also contained Tubificinae—both juvenile and adult—and non-biting midges (Chironomidae). Four weeks after the start of the experiment, in addition to the taxa named above, there were small numbers of dragonflies (Odonata), true bugs (Heteroptera), and gastropods (Gastropoda) (Figure 3).

### 3.3. Dominance Structure

After the first two weeks, the proportions of taxa identified in the control material were as follows: Amphipoda 11%, Coleoptera 8%, Hirudinea 13%, Chironomidae 13%, Tubificinae 16%, Gastropoda 12%, Heteroptera 14%, and Odonata 13%. After four weeks, the proportions of taxa differed slightly from each other and were as follows: Amphipoda 14%, Coleoptera 12%, Hirudinea 15%, Chironomidae 11%, Tubificinae 12%, Gastropoda 16%, Heteroptera 13%, and Odonata 7%. After six weeks, Tubificane (17%), Gastropoda (17%), and Heteroptera (17%) attained the largest share of the fauna in the control sample, followed by Amphipoda (10%), Hirudinea (10%), Odonata (10%), Chironomidae (10%), and Coleoptera (9%). After eight weeks, the proportions of taxa identified in the control material were as follows: Amphipoda 8%, Coleoptera 14%, Hirudinea 8%, Chironomidae 14%, Tubificinae 14%, Gastropoda 17%, Heteroptera 11%, and Odonata 14%.

**Figure 4:**
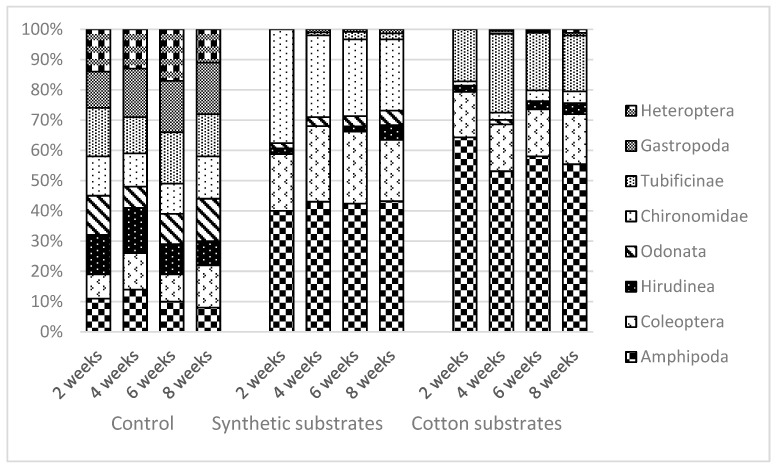
Dominance structure of particular taxonomics group of aquatica invertebrates (% in the total numbers substrate/m^2^ of surfaces) in the different experimental variants.

After the first two weeks of the experiment, amphipods attained the largest share of the fauna on the synthetic material, accounting for 40% of all identified organisms, followed by Tubificinae (37.6%), Coleoptera (18.8%), Hirudinea (18.8%), and Chironomidae (18.8%). After four weeks of exposure, amphipods remained dominant, with a 43% share of the total abundance, followed by Tubificinae (27%), Coleoptera (25%), Chironomidae (3%), Heteroptera (1%), and Gastropoda (1%). After six weeks of exposure, Amphipoda accounted for 42.4% of the organisms colonizing the synthetic fabric, followed by Tubificinae (25.4%) and Coleoptera (23.8%). The remaining taxa accounted for less than 10% of all identified organisms (Chironomidae 3.4%, Hirudinea 1.7%, and Gastropoda 0.8%). After eight weeks, the share of amphipods increased only slightly, reaching 43.4% of all organisms colonizing the material, while Tubificinae accounted for 23.6%, Coleoptera for 20.3%, Hirudinea 4.8%, Chironomidae 4.8%, Gastropoda 3%, and Heteroptera 1.3% (Figure 4).

On the cotton substrates, Amphipoda accounted for 64.3% of organisms, Tubificinae for 17.2%, Coleoptera 15%, Hirudinea 2.1%, and Chironomidae 1.4%. No dragonflies, gastropods, or true bugs were detected. After four weeks of exposure, Amphipoda had a 53.1% share of all identified organisms, Tubificinae 26%, and Coleoptera 15%. Chironomidae (2.4%), Odonata (1.5%), and Gastropoda (0.5%) constituted a small proportion of the fauna. Amphipods were still dominant after six weeks of the experiment (58%), followed by Tubificinae (19%), Coleoptera (15.5%), and Hirudinea (2.8%). Chironomidae accounted for 3.5%, while the combined share of Chironomidae, Gastropoda, and Heteroptera did not exceed 10%. Amphipoda remained the most abundant taxon colonizing the cotton fabric after eight weeks (55.4%), followed by Tubificinae (18.4%) and Coleoptera (16.6%), while Hirudinea (3.6%) and Chironomidae (3.9%) accounted for a small proportion of the fauna (Figure 4).

### 3.4. Statistical Analysis

A significant relationship was noted between the exposure time and the abundance of organisms present on the substrates used in the experiment. The degree of correlation between the abundance of individual taxonomic groups and the exposure time was varied. Kendall’s tau correlation coefficient indicated statistical significance (*p* > 0.05) in the case of four taxa. For Amphipoda, Coleoptera, Tubificinae, and Gastropoda, the null hypothesis (H_0_) indicating the absence of a relationship between exposure time and abundance was rejected in favour of the alternative hypothesis (H_A_). For the remaining taxa, for which *p* < 0.05, H_0_ was accepted. Kendall’s tau correlation coefficient ranged from −0.24 to 0.67 for Odonata, Hirudinea, Chironomidae, and Heteroptera, which indicates that exposure time had a minor (insignificant) effect on the abundance of these organisms.

**Figure 5:**
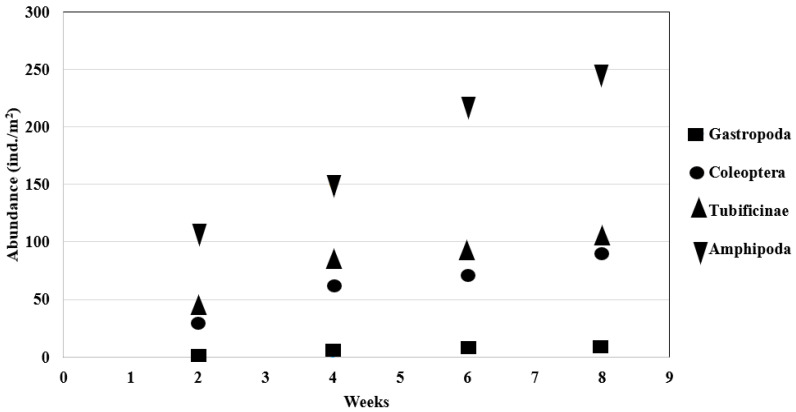
Changes in abundance at successive exposure times.

The correlation coefficient showed that the exposure time had a major influence on the abundance of Amphipoda, Coleoptera, Tubificinae, and Gastropoda. The relationship between the data increases with the value of this coefficient. For Amphipoda, Coleoptera, Tubificinae, and Gastropoda, there was a total positive correlation (Tau-b = 1). The value +1 for the correlation coefficient indicates perfectly correlated data, and thus direct dependence of the parameters. With the passage of exposure time, the abundance of the taxa Amphipoda, Coleoptera, Tubificinae and Gastropoda increased (Table 2, Figure 5).

## 4. Discussion

### 4.1. Effect of the Natural Environment on the Structure of Invertebrate Macrofauna in the Water

Relatively high species richness of aquatic invertebrates was noted in the River Bystrzyca. In both the experiment and the control sample, there were eight taxonomic groups of macroinvertebrates. These results are consistent with the claim by Vannote (1980) that taxonomic diversity in upland rivers is relatively high [23]. Studies conducted in other watercourses of this type also indicate a high density and diversity of macroinvertebrates [24].

The substrates used in the experiment for potential colonization were also habitats for a variety of invertebrate macrofauna. The substrates used in the experiment for potential colonization influenced the diversity of habitat conditions. The cotton items were colonised with much higher numbers of organisms than the synthetic ones. The experiment showed that cotton substrates provide more favourable living conditions for invertebrate macrofauna. This may be due in part to the trophic interactions between various groups of organisms in the aquatic ecosystem, as well as to more favourable food conditions on cotton substrates. Research by Mieczan and Puk (2010) demonstrated that natural substrates are much more readily colonised by organisms such as protozoa and small metazoa, which can be a potential food source for macroinvertebrates [25]. Moreover, insects in an aquatic environment serve as food for nearly all predatory vertebrates and invertebrates, and many of them are predators, such as dragonflies (Odonata). They feed on other small insects or arachnids [3]. Therefore, their presence may have been due to the numerous flies noted in the experiment or the less numerous true bugs and beetles. Another study also showed that macroinvertebrates can be a food source for other aquatic organisms [17]. The colonisation of substrates by aquatic macroinvertebrates is significantly related to time, which may indicate the adaptive capacity of these organisms.

The statistical analysis showed that the abundance of Amphipoda, Coleoptera, Tubificinae, and Gastropoda increased with the exposure time. This is confirmed by the correlation coefficient of +1, which indicates a close relationship between the abundance of macrofauna in the flowing water and exposure time. Studies by Davis (1992) and by Anderson and VanLaerhoven (1996) also confirmed that colonization of substrates by aquatic macroinvertebrates is significantly correlated with time [16,26].

### 4.2. The Role of Invertebrate Macrofauna of the Water Column in Forensic Entomology

The analysed substrates were mainly colonised by amphipods (Amphipoda), dragonflies (Odonata), true bugs (Heteroptera), beetles (Coleoptera), flies (Diptera), leeches (Hirudinea), and gastropods (Gastropoda). Some of these groups play an important role in forensic entomology, i.e., in determining details pertaining to a death and correctly establishing the post-mortem interval [17,27].

Entomological evidence can consist of live or dead insects or traces of their activity. According to Frątczak-Łagiewska (2016), flies (Diptera) and beetles (Coleoptera) are the most important taxa for forensic entomology, due to their embryonic and extraembryonic development [7]. Our experiment showed a fairly high abundance of these taxa on the colonised materials. Flies and beetles undergo complete metamorphosis, whereby the larva develops into the adult form via a pupal stage. When they invade a body, they begin to lay eggs in natural orifices, e.g., in the head or genitals. Although the egg is the shortest stage of development in flies, in some circumstances it can be the only useful entomological evidence for determining the time of death [19,27]. In the field experiment, flies of the family Chironomidae were present in larval form. If eggs laid by insects are present on the body in its early stages of decomposition and in areas other than natural orifices, this may indicate that the person died as a result of inflicted wounds. In addition, beetles and non-biting midges are the first to colonise a corpse in the initial stages of succession, and their current state of development is a source of knowledge about the length of exposure of the body and about climate conditions [10,28]. The results of the experiment, together with literature data, suggest that information regarding the abundance and anatomical features of flies and beetles can serve as evidence in an investigation [19,27,28]. Other organisms colonizing corpses and other substrates submerged in water at an early stage of succession include blow flies (Calliphoridae), carrion beetles (Silphidae), and skin beetles (Dermestidae). These taxa, however, were not detected in the experiment.

The experiment did reveal the presence of dragonflies (Odonata). These taxa are often found near water bodies, and thus they can be used in forensic entomology. Dragonflies can feed on small arachnids or insects colonizing a body, including flies, which were found in fairly large numbers in the experiment [17]. Therefore, their presence on the colonised material may be closely linked to migration in search of food. This is supported by information on adult and larval water nymphs [17]. According to literature data, these organisms are used to estimate the time elapsed after submersion or drowning, and thus they can provide a great deal of information about a suspected crime [3,10].

The genus *Gammarus* is not a taxon widely used in forensic entomology. Research conducted in Italy demonstrated that the crustacean *Gammarus pulex*, in contact with a body submerged in water, leaves numerous small holes in the skin [3]. Characteristic punctures may be traces of the activity of amphipods on the body, which indicates that a body submerged in water may be colonised by Amphipoda [19]. It can be concluded from the data obtained in our experiment that amphipods can provide important information about a suspected crime. However, their broad range of applications extends beyond the field of forensic entomology [10,19].

The substrates were also colonised by true bugs (Heteroptera). This is not a taxon widely used in forensic entomology, but it is found among the fauna of submerged corpses [10,19]. In addition, the experiment revealed the presence of ringed worms (Annelida) and gastropods (Gastropoda). Literature sources indicate that these taxa are not used in forensic entomology and that they are not closely associated with the fauna of submerged corpses [10]. The presence of leeches and gastropods may have been associated with their colonization of the materials, which could potentially serve as evidence, in the search for shelter or food [17].

## 5. Conclusions

The experiment revealed the presence of three major taxa (arthropods, insects, and annelids), which were much more abundant on the cotton substrates than on the synthetic ones. The abundance of aquatic macrofauna on the exposed items increased in direct proportion to the duration of the experiment, which may indicate the adaptability of these organisms to new habitat conditions. The results of the study may be useful in evidentiary hearings. Flies (Diptera), beetles (Coleoptera), and dragonflies (Odonata) are invertebrate macrofauna used in forensic entomology. The other taxa, though not widely used in court proceedings, may also provide valuable information about the circumstances of an incident. It seems that amphipods in particular are an underappreciated group that could help to clear up discrepancies and ultimately play a role in solving a criminal case. In criminal procedure, doubts are resolved in favour of the accused, but the better the evidence is preserved and analysed, the fewer doubts will remain, and thus probability becomes certainty as to guilt or the absence of guilt.

## Figures and Tables

**Table 1:** Experimental design.

Artificial Substrates Samples		Control Samples
12 Socks (Synthetic)	12 Shirts (Cotton)	Bottom Sediments (98 cm^2^) from River (12 Samples)
**Start of the Experiment**		
**2 Weeks**		
3 Socks	3 Shirts	3 Samples
**4 Weeks**		
3 Socks	3 Shirts	3 Samples
**6 Weeks**		
3 Socks	3 Shirts	3 Samples
**8 Weeks**		
3 Socks	3 Shirts	3 Samples
**Completion of the experiment**		

**Table 2:** Kendall’s tau-b correlation coefficient: exposure time vs macroinvertebrate abundance *Tau-b*—Kendall’s tau-b correlation coefficient, *p*—*p*-Value).

Macroinvertebrates	Exposure Time	
	*Tau-b*	*p*
Amphipoda	1.00	0.01
Coleoptera	1.00	0.01
Hirudinea	0.67	0.17
Odonata	−0.24	0.65
Chironomidae	0.67	0.17
Tubificinae	1.00	0.01
Gastropoda	1.00	0.01
Heteroptera	0.67	0.17

## Data Availability

Not applicable.
